# Downregulation of miR-155-5p enhances the anti-tumor effect of cetuximab on triple-negative breast cancer cells via inducing cell apoptosis and pyroptosis

**DOI:** 10.18632/aging.103669

**Published:** 2021-01-05

**Authors:** Wen Xu, Changfeng Song, Xiaotong Wang, Yueqi Li, Xue Bai, Xin Liang, Jingjing Wu, Jianwen Liu

**Affiliations:** 1 State Key Laboratory of Bioreactor Engineering and Shanghai Key Laboratory of New Drug Design, School of Pharmacy, East China University of Science and Technology, Shanghai 200237, P.R. China; 2 Department of Breast, Longhua Hospital Affiliated to Shanghai University of TCM, Shanghai 200032, P.R. China

**Keywords:** triple-negative breast cancer, microRNA 155-5p, cetuximab, apoptosis, pyroptosis

## Abstract

Cetuximab resistance is the main obstacle for the treatment of EGFR overexpression cancer, including triple-negative breast cancer (TNBC). MicroRNA (miRNA)-155-5p is upregulated in TNBC cells; thus, the present study explored whether the downregulation of miR-155-5p enhanced the anti-tumor effect of cetuximab in TNBC cells. MDA-MB-231 and MDA-MB-468 cells were infected with lentivirus-epidermal growth factor receptor (EGFR) for 72 h to obtain EGFR-overexpressed cell lines (MDA-MB-231 and MDA-MB-468). The inhibitory effects of cetuximab on the proliferation and migration of EGFR-overexpressed MDA-MB-468 cells were enhanced following transfection with the miR-155-5p antagomir, and miR-155-5p knockdown enhanced the pro-apoptotic effect of cetuximab on EGFR-overexpressed MDA-MB-468 cells. Further, the luciferase reporter assay revealed that gasdermin E (GSDME) was the direct binding target of miR-155-5p. The combination of cetuximab with the miR-155-5p antagomir promoted pyroptosis in EGFR-overexpressed MDA-MB-468 cells via the upregulation of GSDME-N and cleaved caspase-1. Results from the *in vivo* experiments confirmed that the downregulation of miR-155-5p enhanced the anti-tumor effect of cetuximab in an MDA-MB-468 xenograft model and on EGFR-overexpressed TNBC cells via inducing cell apoptosis and pyroptosis. Therefore, cetuximab combination with an miR-155-5p antagomir may be a novel therapeutic strategy for the treatment of TNBC.

## INTRODUCTION

Breast cancer is the most common malignancy in females and remains a major public health issue worldwide [1]. Approximately 1.4 million new cases were diagnosed in 2008, and the incidence of breast cancer remains alarmingly high [2]. Evidence from studies suggest that that obesity, alcohol intake, a late age at first birth, and late menopause are risk factors for the development of breast cancer [3]. In addition, the 5-year survival rate of patients with breast cancer in developed countries is 80%, whereas the 5-year survival rate in developing countries is as low as 40% [2]. Triple-negative breast cancer (TNBC) is the most aggressive subtype of breast cancer that is characterized by a deficiency in the estrogen receptor (ER), progesterone receptor (PR), and human epidermal growth factor receptor 2 (HER2) [4]. Therefore, the identification of novel therapeutic targets and approaches may improve the prognosis and treatment of TNBC.

Cetuximab is a monoclonal antibody that belongs to a class of molecular targeted therapy drugs that target the epidermal growth factor receptor (EGFR) [5, 6]. Specifically, cetuximab binds to the extracellular domain of EGFR and suppresses the activation of a series of downstream signaling pathways [7]. Results from previous studies demonstrated that the expression of EGFR was increased in patients with TNBC [8, 9]. Liao et al indicated that cetuximab treatment could induce apoptosis and inhibit growth of the EGFR-expressed TNBC cells *in vitro* and *in vivo* [10]. Therefore, cetuximab is an effective treatment for some patients with breast cancer. However, a large percentage of patients with breast cancer are resistant to anti-EGFR therapies after long period of treatment with EGFR inhibitor [11]. Therefore, novel therapies for the treatment of TNBC are needed.

MicroRNAs (miRNAs) are a class of endogenous noncoding single-stranded RNA molecules that contain 18-24 nucleotides [12]. MiRNAs regulate post-transcriptional gene expression by binding to the complementary sequences in the 3’-untranslated region (3’-UTR) of their target mRNAs [13]. Recently, miRNAs have emerged as novel biomarkers for various cancers, including breast cancer [14]. Liu et. al. [15] found that the level of miR-155-3p was up-regulated in breast cancer cells. Results from another study revealed that miR-155 promoted the proliferation of breast cancer cells and suppressed apoptosis in breast cancer cells [16].

In this study, we identified GSDME harbored a conserved miR-155-5p cognate sites using TargetScan bioinformatics tool, and predicted that GSDME was a potential target of miR-155-5p. GSDME was identified as the executioner of pyroptosis [17]. Pyroptosis is a novel form of programmed necrosis, which is triggered upon formation of caspase-1-activating inflammasomes [18]. Active caspase-1 can lead to increased production of gasdermin D and proinflammatory cytokines IL-1β and IL-18 [17]. Therefore, this study investigated whether the downregulation of miR-155-5p enhanced the anti-tumor effect of cetuximab in TNBC cells via targeting GSDME in order to provide an alternative therapeutic option for patients with TNBC.

## RESULTS

### EGFR is overexpressed in TNBC cells

**Figure 1:**
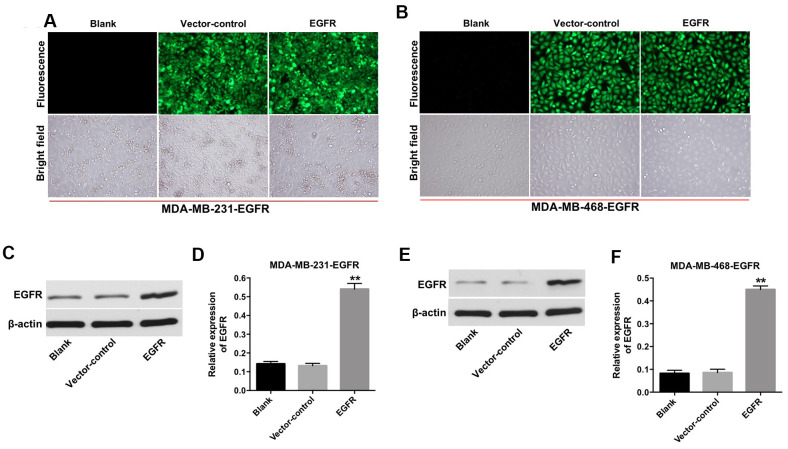
**Overexpression of EGFR in TNBC cells.** (**A**) MDA-MB-231 (**B**) and MDA-MB-468 cells were transfected with lenti-EGFR for 72 h. The transfection efficacy of the cells was observed under a fluorescent microscope (×200 magnification). (**C**–**F**) The expression of EGFR in MDA-MB-231 and MDA-MB-468 cells was analyzed by Western blotting. **P < 0.01 compared with the vector-control group.

First, we established TNBC cell lines (e.g., MDA-MB-231 and MDA-MB-468) with stable EGFR overexpression. As shown in Figure 1A and 1B, the fluorescent expression confirmed that the MDA-MB-231 and MDA-MB-468 cells were effectively transfected with the lentivirus after incubation for 72 h. In addition, the results from the quantitative real-time polymerase chain reaction (qRT-PCR) assay indicated that the expression of EGFR was significantly increased in MDA-MB-231 and MDA-MB-468 cells following transfection with lentivirus-EGFR (Figure 1C–1F). These findings indicated that EGFR was overexpressed in the MDA-MB-231 and MDA-MB-468 cells.

### Downregulation of miR-155-5p enhanced the anti-proliferative effect of cetuximab in TNBC cells

**Figure 2:**
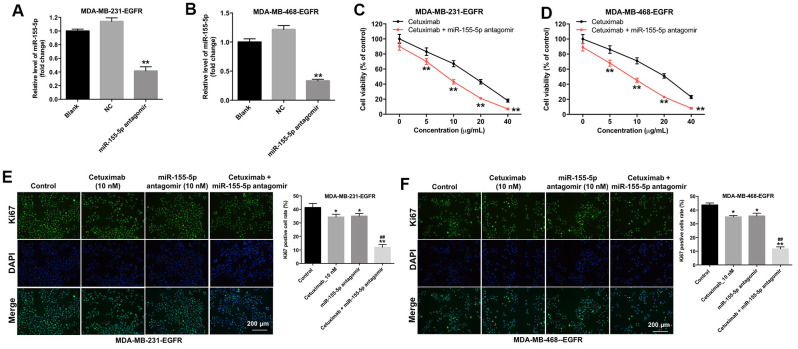
**Downregulation of miR-155-5p enhances the anti-proliferative effect of cetuximab in TNBC cells.** (**A**) EGFR-overexpressed MDA-MB-231 and (**B**) MDA-MB-468 cells were transfected with miR-155-5p for 72 h. RT-qPCR was used to detect the level of miR-155-5p in cells. (**C**) EGFR-overexpressed MDA-MB-231 and (**D**) MDA-MB-468 cells were treated with cetuximab (0, 5, 10, 20, or 40 nM) and/or the miR-155-5p antagomir (10 nM) for 72 h. The Cell Counting Kit-8 (CCK-8) assay was used to detect cell viability. (**E**) EGFR-overexpressed MDA-MB-231 and (**F**) MDA-MB-468 cells were treated with cetuximab (10 nM) and/or the miR-155-5p antagomir (10 nM) for 72 h. Relative fluorescence expression levels were quantified by Ki67 and DAPI staining. **P < 0.01 compared with the control group. ^##^P < 0.01 compared with the cetuximab 10 nM group.

To determine the effect of miR-155-5p on the proliferation of MDA-MB-231 and MDA-MB-468 cells, we transfected the MDA-MB-231 and MDA-MB-468 cells with an miR-155-5p antagomir. As shown in Figure 2A and 2B, the level of miR-155-5p was markedly downregulated in the EGFR-overexpressed MDA-MB-231 and MDA-MB-468 cells following transfection with the miR-155-5p antagomir. In addition, cetuximab inhibited the viability of the EGFR-overexpressed MDA-MB-231 and MDA-MB-468 cells in a dose-dependent manner (Figure 2C and 2D). The downregulation of miR-155-5p enhanced the cytotoxic effect of cetuximab in EGFR-overexpressed MDA-MB-231 and MDA-MB-468 cells (Figure 2C and 2D). In addition, the IC50 value of cetuximab was 16.01 μg/mL and 20.08 μg/mL in EGFR-overexpressed MDA-MB-231 and MDA-MB-468 cells, respectively. When cetuximab was combined with miR-155-5p antagomir (10 nM), the IC50 value of cetuximab was decreased to 7.51 μg/mL and 9.19 μg/mL in EGFR-overexpressed MDA-MB-231 and MDA-MB-468 cells, respectively. Furthermore, the CI value of cetuximab combined with miR-155-5p antagomir in EGFR-overexpressed MDA-MB-231 and MDA-MB-468 cells were less than 0.9, which indicated the synergism effect (Table 1). These results suggested that combination of cetuximab with miR-155-5p antagomir synergistically inhibited the proliferation of EGFR-overexpressed MDA-MB-231 and MDA-MB-468 cells.

**Table 1:** Evaluation of combination of cetuximab with miR-155-5p antagomir in MDA-MB-231 and MDA-MB-468 cells (72 h treatment).

**Drug combination**	**MDA-MB-231 cells**		**MDA-MB-468 cells**	
	**IC 50 value**	**CI values**	**IC 50 value**	**CI values**
cetuximab (range 0 from 40 μg/mL)	16.01 μg/mL	-	20.08 μg/mL	-
cetuximab + 10 nM miR-155-5p antagomir	7.51 μg/mL	0.73	9.19 μg/mL	0.81

Results from immunofluorescence staining assay demonstrated that the proliferation of EGFR-overexpressed MDA-MB-231 and MDA-MB-468 cells was slightly inhibited following treatment with cetuximab or the miR-155-5p antagomir as compared with the proliferation of cells in the control group (Figure 2E and 2F). Additionally, the proliferation of EGFR-overexpressed MDA-MB-231 and MDA-MB-468 cells was significantly inhibited in the cetuximab and miR-155-5p treatment group as compared with that in the cetuximab treatment group (Figure 2E and 2F). These results suggested that the downregulation of miR-155-5p enhanced the anti-proliferative effect of cetuximab in EGFR-overexpressed TNBC cells.

### Downregulation of miR-155-5p enhanced the cytotoxicity effect of cetuximab in TNBC cells

**Figure 3:**
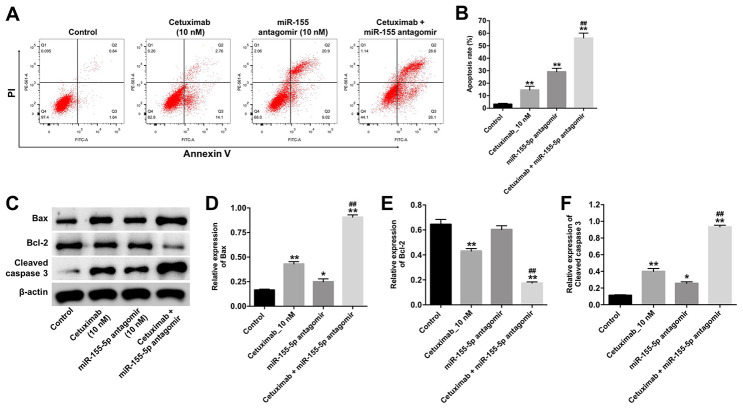
**Downregulation of miR-155-5p enhances the pro-apoptotic effect of cetuximab in TNBC cells.** EGFR-overexpressed MDA-MB-468 cells were treated with cetuximab (10 nM) and/or the miR-155-5p antagomir (10 nM) for 72 h. (**A**, **B**) Apoptotic cells were detected by flow cytometry. (**C**) The expression levels of Bax, Bcl-2, and cleaved caspase-3 in cells were detected by Western blotting. (**D**–**F**) The relative expressions of Bax, Bcl-2, and cleaved caspase-3 were normalized to β-actin. *P < 0.05, **P < 0.01 compared with the control group. ^##^P < 0.01 compared with the cetuximab 10 nM group.

Next, the cytotoxicity effect of cetuximab combined with the miR-155-5p antagomir on EGFR-overexpressed MDA-MB-468 cells was measured with flow cytometry, which suggested the forms of death including apoptosis (in Q2 and Q3 quadrant), necrosis or pyroptosis (in Q1 and Q2 quadrant). As indicated in Figure 3A and 3B, 10 nM cetuximab markedly induced the apoptosis of EGFR-overexpressed MDA-MB-468 cells, and the downregulated miR-155-5p significantly enhanced the pro-necrosis or pro-pyroptosis effect in EGFR-over-expressed TNBC cells. In addition, the expression of Bax and cleaved caspase-3 were increased in EGFR-overexpressed MDA-MB-468 cells in the cetuximab and miR-155-5p antagomir treatment group as compared with that in the cetuximab treatment group. However, the level of BCl-2 was reduced in EGFR-overexpressed MDA-MB-468 cells in the cetuximab and miR-155-5p treatment group as compared with that in the cetuximab treatment group (Figure 3C–3F). These data illustrated that the downregulation of miR-155-5p enhanced the cytotoxicity effect of cetuximab in EGFR-overexpressed MDA-MB-468 cells, however, the type of death need be identified by other assays.

### Downregulation of miR-155-5p enhanced the inhibitory effect of cetuximab on the migration and invasion of TNBC cells

**Figure 4:**
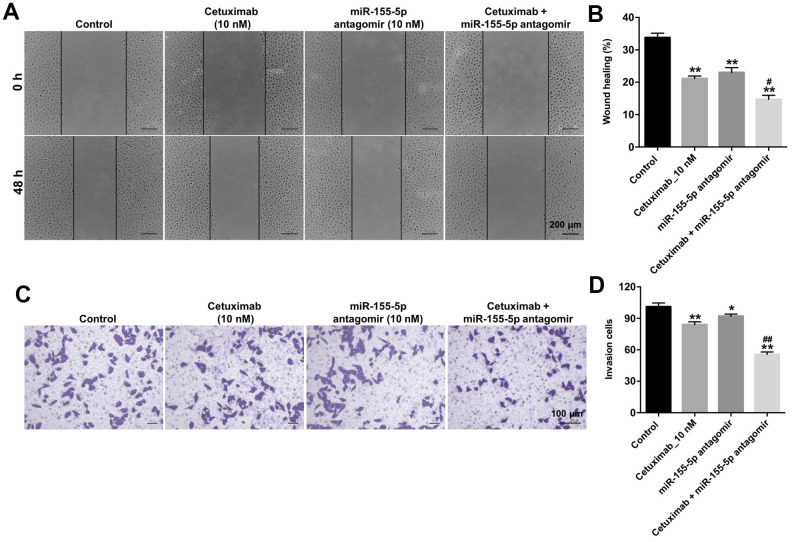
**Downregulation of miR-155-5p enhances the inhibitory effect of cetuximab on the migration and invasion of TNBC cells.** (**A**, **B**) EGFR-overexpressed MDA-MB-468 cells were treated with cetuximab (10 nM) and/or the miR-155-5p antagomir (10 nM) for 48 h. Cell migration was detected using the wound healing assay. (**C**, **D**) EGFR-overexpressed MDA-MB-468 cells were treated with cetuximab (10 nM) and/or the miR-155-5p antagomir (10 nM) for 24 h. Cell invasion was detected using the transwell invasion assay. *P < 0.05, **P < 0.01 compared with the control group. ^##^P < 0.01 compared with the cetuximab 10 nM group.

We performed wound healing and transwell invasion assays to determine the effect of cetuximab and the miR-155-5p antagomir on the migration and invasion of EGFR-overexpressed TNBC cells. As shown in Figure 4A–4D, cetuximab or the miR-155-5p antagomir slightly inhibited the migration and invasion abilities of EGFR-overexpressed MDA-MB-468 cells. However, the migration and invasion abilities of EGFR-overexpressed MDA-MB-468 cells in the cetuximab and miR-155-5p antagomir treatment group were suppressed as compared with those in the cetuximab treatment group (Figure 4A–4D). These results indicated that the downregulation of miR-155-5p enhanced the inhibitory effect of cetuximab on the migration and invasion of EGFR-overexpressed MDA-MB-468 cells.

### GSDME was a direct binding target of miR-155-5p

**Figure 5:**
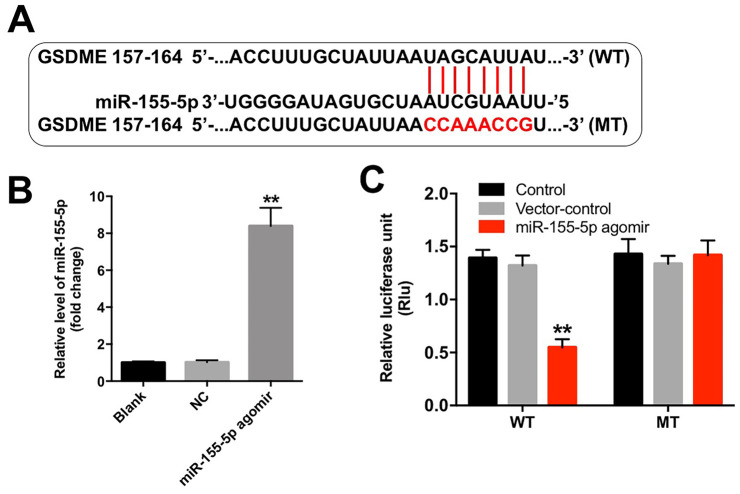
**miR-155-5p directly binds to GSDME.** (**A**) The 3'-UTR of GSDME harbors the miR-155-5p cognate sites. (**B**) Cells that were transfected with the miR-155-5p agomir were detected with RT-qPCR. (**C**) The relative luciferase activity of the reporter plasmids carrying the WT- or MT-GSDME 3'-UTR in EGFR-overexpressed MDA-MB-468 cells following co-transfection with the miR-155-5p agomir was detected using the dual luciferase reporter assay. **P < 0.01 compared with the control group.

Although various target genes of miR-155-5p were obtained from the TargetScan database (http://www.targetscan.org/vert_71/), GSDME, a marker of pyroptosis, was used as the predicted target gene of miR-155-5p in the current study (Figure 5A). In addition, the level of miR-155-5p was significantly upregulated following transfection with the miR-155-5p agomir (Figure 5B). Furthermore, the results from the dual luciferase reporter assay confirmed that luciferase activity was reduced in the EGFR-overexpressed MDA-MB-468 cells following co-transfection with the WT-GSDME segment and miR-155-5p agomir as compared with that in the vector-control group (Figure 5C). These results indicate that GSDME is a direct binding target of miR-155-5p.

### Cetuximab combined with the miR-155-5p antagomir induced pyroptosis in TNBC cells

**Figure 6:**
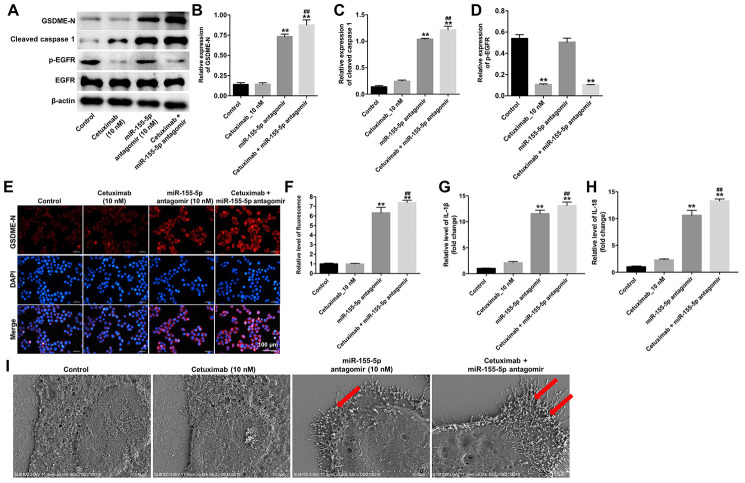
**Cetuximab and miR-155-5p antagomir combination treatment induces pyroptosis in TNBC cells.** EGFR-overexpressed MDA-MB-468 cells were treated with cetuximab (10 nM) and/or the miR-155-5p antagomir (10 nM) for 72 h. (**A**) Expression levels of GSDME-N, cleaved caspase-1, and p-EGFR in cells were detected by western blotting. (**B**, **C**) The relative expression levels of GSDME-N and cleaved caspase-1 were normalized to β-actin. (**D**) The relative expression level of p-EGFR in cells was normalized to EGFR. (**E**, **F**) The relative fluorescence expression levels were quantified by GSDME-N and DAPI staining. (**G**, **H**) Levels of IL-1β and IL-18 in cells were detected by RT-qPCR. (**I**) The ultrastructure of the cells was observed under a transmission electron microscope (TEM). Red arrowheads indicate the large bubbles emerging from the plasma membrane. **P < 0.01 compared with the control group. ^##^P < 0.01 compared with cetuximab the 10 nM group.

Results from previous studies indicate that the N-terminus of GSDME trans-locates to the cell membrane and induces cell pyroptosis [19]. To identify the mechanism underlying miR-155-5p-regulated pyroptosis in EGFR-overexpressed MDA-MB-468 cells, the protein levels of GSDME-N, cleaved caspase-1, and p-EGFR were detected by Western blotting assays. Results from the Wester blotting analysis revealed that cetuximab had no effect on the expression of GSDME-N and cleaved caspase-1 in EGFR-overexpressed MDA-MB-468 cells; however, the miR-155-5p antagomir significantly increased the levels of GSDME-N and cleaved caspase-1 in the EGFR-overexpressed MDA-MB-468 cells (Figure 6A–6C). Cetuximab markedly inhibited the expression of p-EGFR in EGFR-overexpressed MDA-MB-468 cells, whereas treatment with the miR-155-5p antagomir had no effect on the expression of p-EGFR in the EGFR-overexpressed MDA-MB-468 cells (Figure 6A and 6D). Moreover, results from the immunofluorescence staining assay confirmed that cetuximab combined with the miR-155-5p antagomir increased the level of GSDME-N in EGFR-overexpressed MDA-MB-468 cells (Figure 6E and 6F). Meanwhile, results from the RT-qPCR analysis revealed that cetuximab had no effect on the levels of IL-1β and IL-18 in EGFR-overexpressed MDA-MB-468 cells; however, the miR-155-5p antagomir markedly increased the levels of IL-1β and IL-18 in the EGFR-overexpressed MDA-MB-468 cells (Figure 6G and 6H). Morphologically, cells in the miR-155-5p antagomir and cetuximab + miR-155-5p antagomir group exhibited the characteristic pyroptotic phenotype (e.g., cell swelling and large bubbles emerging from the plasma membrane) (Figure 6I). These data indicated that cetuximab combined with the miR-155-5p antagomir induced pyroptosis in EGFR-overexpressed TNBC cells.

### Downregulation of miR-155-5p enhanced the anti-tumor effect of cetuximab in TNBC cells *in vivo*

**Figure 7:**
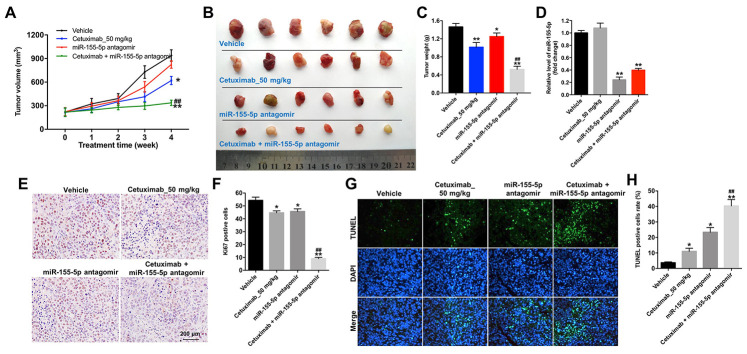
**Downregulation of miR-155-5p enhances the anti-tumor effect of cetuximab in TNBC cells *in vivo.* EGFR-overexpressed MDA-MB-468 cells were subcutaneously injected into nude mice.** (**A**) Tumor volumes of the mice were measured weekly. (**B**) The tumors were excised from xenografts and imaged on day 28. (**C**) The tumor weights were calculated. (**D**) RT-qPCR was used to determine the level of miR-155-5p in the tumor tissues. (**E**, **F**) The number of Ki67 positive cells in tumor tissues was measured using an immunohistochemistry (IHC) analysis. (**G**, **H**) The cell apoptosis in tumor tissues was measured using a TUNEL analysis. *P < 0.05, **P < 0.01 compared with the vehicle group. ^##^P < 0.01 compared with the cetuximab 10 nM group.

**Figure 8:**
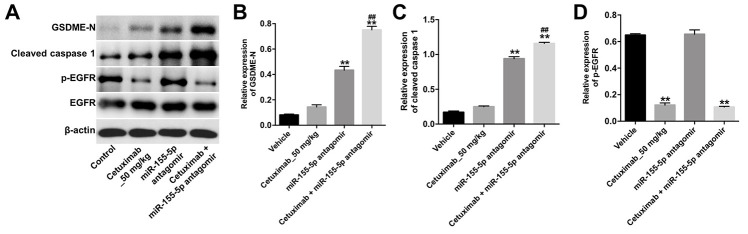
**Downregulation of miR-155-5p enhances the anti-tumor effect of cetuximab in TNBC cells *in vivo* via inducing pyroptosis.** (**A**) The expression levels of GSDME-N, cleaved caspase-1 and p-EGFR in tumor tissues were detected by Western blotting. (**B**, **C**) The relative expression levels of GSDME-N and cleaved caspase-1 in tumor tissues were normalized to β-actin. (**D**) The relative expression of p-EGFR in tumor tissues was normalized to EGFR. **P < 0.01 compared with the vehicle group. ^##^P < 0.01 compared with the cetuximab 10 nM group.

We further investigated the role of cetuximab combined with the miR-155-5p antagomir in a MDA-MB-468 xenograft mouse model. As shown in Figure 7A–7C, the tumor volume and tumor weight were decreased in the cetuximab + miR-155-5p antagomir treatment group as compared with those in the cetuximab treatment group. In addition, treatment with the miR-155-5p antagomir or combination treatment notably decreased the level of miR-155-5p in tumor tissues (Figure 7D). Moreover, results from the immunohistochemistry (IHC) assay indicated that combination treatment markedly suppressed cell proliferation in tumor tissues (Figure 7E and 7F). Meanwhile, results from the TUNEL assay revealed that combination treatment notably induced cellapoptosis in tumor tissues (Figure 7G and 7H). Combination treatment upregulated the expression of GSDME-N and cleaved caspase-1 in tumor tissues, but downregulated the expression of p-EGFR (Figure 8A–8D). These data indicated that the downregulation of miR-155-5p enhanced the anti-tumor effect of cetuximab in TNBC cells *in vivo* via inducing pyroptosis.

## DISCUSSION

EGFR is overexpressed in TNBC cells; therefore it is a potential target for anticancer drugs [10]. Results from a study conducted by Nakamura et. al. [20] revealed that EGFR, which is a therapeutic target for oral cancer, was overexpressed in oral cancer cells. Cetuximab exhibits anti-tumor effects in human cancers via targeting EGFR [20–22]. In this study, we confirmed that cetuximab suppressed the proliferation of EGFR-overexpressed TNBC cells *in vitro* and *in vivo* via inhibiting the activation of EGFR.

Recently, miRNAs have gained attention as novel targets for the treatment of breast cancer [23]. Hong et al [24] indicated that miR-7 sensitized breast cancer cells to paclitaxel and carboplatin, while Shi et al [25] found that the overexpression of miR-129-5p enhanced the anti-tumor effect of taxol in breast cancer cells. Studies have also demonstrated that miR-155 functioned as an oncogene in several cancers, such as oral squamous cell carcinoma, bladder cancer, and anaplastic thyroid cancer [26–28]. In this study, we found that miR-155-5p antagomir or cetuximab slightly inhibited the proliferation of EGFR-overexpressed TNBC cells. However, the complete knockdown of miR-155-5p markedly enhanced the anti-proliferative effect of cetuximab in EGFR- overexpressed TNBC cells *in vitro* and *in vivo*. Moreover, the upregulation of miR-155-5p enhanced the anti-apoptotic effect of cetuximab in EGFR-overexpressed MDA-MB-468 cells via upregulating the expressions of Bax and cleaved caspase-3 and downregulating the expression of Bcl-2. These data suggest that the downregulation of miR-155-5p enhances the anti-tumor effect of cetuximab in EGFR-overexpressed TNBC cells via inducing apoptosis.

To further investigate the molecular mechanisms underlying the miR-155-5p-mediated growth of EGFR-overexpressed TNBC cells, we performed TargetScan and luciferase reporter assays to predict and confirm potential binding targets of miR-155-5p. Results from these assays indicated that GSDME was a potential binding target of miR-155-5p. GSDME is a member of the gasdermin family, which participates in the activation of pyroptosis [29]. Pyroptosis is a type of programmed cell death that is mediated by caspase-1 [30]. Specifically, activated caspase-1 cleaves GSDME into two fragments: N- and C-terminal domains [31]. The N-terminus of GSDME trans-locates into the cell membrane where it induces cell pyroptosis [19, 29]. Pyroptosis is characterized by rapid plasma-membrane rupture and the release of proinflammatory intracellular contents [32]. In this study, we found that the downregulation of miR-155-5p induced pyroptosis of EGFR-overexpressed MDA-MB-468 cells via the upregulation of GSDME, cleaved caspase-1, IL-1β and IL-18. However, cetuximab had no effects on the levels of GSDME-N, cleaved caspase-1, IL-1β and IL-18 in EGFR-overexpressed MDA-MB-468 cells. Thus, although cetuximab exerted an anti-tumor effect in EGFR-overexpressed MDA-MB-468 cells, this effect was not accomplished by inducing pyroptosis. Collectively, the downregulation of miR-155-5p may enhance the anti- tumor effect of cetuximab in EGFR-overexpressed TNBC cells *in vitro* and *in vivo* by inducing pyroptosis.

Apoptosis is the most widely recognized programmed process that lead to non-inflammatory cell death [32]. Meanwhile, pyroptosis (also known as caspase 1-dependent programmed cell death) is another programmed cell death process, and is inherently inflammatory [33]. Our data found that combination treatment suppressed the growth of EGFR-overexpressed TNBC cells *in vitro* and *in vivo* by inducing apoptosis and pyroptosis. However, it is not clear at this point which one (apoptosis or pyroptosis) plays the leading role in triggering cell death in EGFR-overexpressed TNBC cells. Therefore, further study is needed to elucidate the exact mechanism of combination-mediated cell death in TNBC.

## CONCLUSION

In this study, we found that cetuximab exerted anti-tumor effect in EGFR-overexpressed MDA-MB-468 cells via the inhibition of EGFR. In addition, the downregulation of miR-155-5p enhanced the anti- tumor effect of cetuximab in EGFR-overexpressed TNBC cells via inducing apoptosis and pyroptosis. Therefore, the combination of the miR-155-5p antagomir with cetuximab may be an alternative therapeutic approach for the treatment of TNBC.

## MATERIALS AND METHODS

### Cell culture

The human TNBC cell lines (MDA-MB-231, MDA-MB-468) were obtained from Type Culture Collection of the Chinese Academy of Sciences (Shanghai, China). The cells were cultured in DMEM (Thermo Fisher Scientific, Waltham, MA, USA) supplemented with 10% heat-inactivated fetal bovine serum (FBS) and antibiotics (100 U/mL penicillin and 100 mg/mL streptomycin) and incubated at 37 °C in a humidified atmosphere with 5% CO_2_.

### Lentivirus production and cell infection

The EGFR sequence was synthesized by GenePharma and cloned into the lentiviral expression vector. Next, the lenti-EGFR plasmids were infected into 293T cells and incubated for 48 h at 32 °C. After incubation, the supernatant containing the virus particles were collected, and the MDA-MB-231 and MDA-MB-468 cells were seeded into 60-mm cell plates at a density of 4 x 10^5^ cells/well and cultured overnight. The following day, the lenti-EGFR supernatant was added directly to the MDA-MB-231 and MDA-MB-468 cells. Finally, the cells were treated with Puromycin (2.5 μg/mL, Sigma Aldrich) for three days to select stable EGFR overexpressed cell lines (MDA-MB-231, MDA-MB-468).

### Cell transfection

The miR-155-5p agomir (5’-UUAAUGCUAAUCGUGAUAGGGGU-3’) and miR-155-5p antagomir (5’-ACCCCUAUCACGAUUAGCAUUAA-3’) and negative control (NC)were synthesized and purchased from RiboBio (Guangzhou, China) with a stock concentration of 20 μM. Then, 10 nM miR-155-5p antagomir and NC were transfected into the EGFR-overexpressed MDA-MB-231 or EGFR-overexpressed MDA-MB-468 cells using Lipofectamine 2000 (Thermo Fisher Scientific) according to the manufacturer’s protocol.

### Quantitative real-time polymerase chain reaction

Total RNA was extracted using Trizol reagent (Thermo Fisher Scientific) according to the manufacturer’s protocol. The MiScript Reverse Transcription Kit (QIAGEN, Dusseldorf, Germany) was used to synthesize complementary DNA. Real-time PCR was performed in the ABI 7500 Fast Real-Time PCR System (Applied Biosystems) with the SYBR premix Ex Taq II kit (TaKaRa, Dalian, China). The relative level of miR-155-5p was normalized to the internal control (e.g., U6) using the comparative delta CT ^(2-ΔΔCT)^ method. The miR-155-5p and U6 primers were as follows: mR-155 Forward: 5’-TAATGCTAATCGTGATAGGGGTTC-3’; Reverse: 5’-CTCAACTGGTGTCGTGGAGTC-3’. U6: Forward: 5’-CTCGCTTCGGCAGCACAT-3’; Reverse: 5’-AACGCTTCACGAATTTGCGT-3’.

### CCK-8 assay

EGFR-overexpressed MDA-MB-231 or MDA-MB-468 cells were seeded into 96-well plates (1 × 10^5^ cells/well) and incubated overnight. The following day, the cells were treated with cetuximab for 72 h at the following concentrations: 0, 5, 10, 20, or 40 nM. Cell viability was determined using the Cell Counting Kit- 8 (CCK-8) assay (Dojindo, Kumamoto, Japan), and the absorbance of the cells was measured at 450 nm using a microplate reader (Bio-Tek, Winooski, VT, USA).

### Combination studies

The combination index (CI) was used to determine the drug combination studies by using Chou–Talalay method [34]. EGFR-overexpressed MDA-MB-231 or MDA-MB-468 cells were exposed to solutions containing 0, 5, 10, 20, or 40 μg/ml cetuximab combined with miR-155-5p agomir (10 nM). The CI value for the combination of cetuximab and miR-155-5p agomir in TNBC can be described as CI=DA/ICx,A+DB/ICx,B. CI < 0.9, indicates synergistic activity; 0.9 ≤ CI ≤ 1.1, indicates additivity; CI > 1.1 indicates antagonism.

### Immunofluorescence staining assay

Cells were washed twice with PBS, pre-fixed with 4% paraformaldehyde at room temperature for 10 min, and post-fixed with pre-chilled methanol at -20 °C. Next, the cells were incubated with the following primary antibodies: anti-Ki67 and anti-GSDME-N at 4 °C overnight. The following day, the cells were incubated with secondary antibodies at 37 °C for 1 h. All of the antibodies were purchased from Abcam (Cambridge, MA, USA), and the cells were observed under a fluorescent microscope (Olympus BX53 Tokyo, Japan).

### Flow cytometry

Cells were washed twice with pre-chilled PBS and resuspended in a binding buffer. Next, the cells were stained with 5 μL of Annexin V-FITC and 5 μL of propidium Iodide (PI, Thermo Fisher Scientific) for 30 min in the dark according to the protocol. A flow cytometer (BD FACSCanto II, BD Bioscience, Franklin Lake, NJ, USA) was used to determine the number of annexin V-FITC-positive apoptotic cells.

### Wound healing assay

Cells (2 × 10^5^ cells/well) were seeded into a 12-well culture plate and cultured in DMEM supplemented with 10% FBS. Once the cells reached 80 % confluency, a wound area was generated with a sterile 200 μL pipette tip, and the cells were washed with PBS to remove non-adherent cells. Next, the cells were treated with cetuximab (10 nM) and/or miR-155-5p antagomir (10 nM) for 48 h. Finally, the wound closure was photographed at 0 and 48 h in five random microscopic regions using a fluorescent microscope (Olympus).

### Transwell invasion assay

A 24-well transwell (Corning New York, NY, USA) was pre-coated with Matrigel (BD Bioscience, Franklin Lake, NJ, USA). Cells (5 × 10^4^ cells) that were suspended in serum-free DMEM medium were seeded into the upper chamber, and 600 μL of DMEM supplemented with 10% FBS were added to the lower chamber to induce cell invasion. After 24 h of incubation, the cells that attached to the lower surface of the chamber were stained with 0.2% crystal violet. Finally, the cells were photographed at 0 and 48 h in five random microscopic regions using a fluorescent microscope (Olympus).

### Western blot assay

Protein concentrations were determined using the BCA protein assay kit (Beyotime, Beijing, China). The protein samples were separated on 10% sodium dodecyl sulfate (SDS) polyacrylamide gels and transferred onto polyvinylidene fluoride (PVDF) membranes (Millipore, Billerica, MA, USA). Next, the membranes were blocked with 5% non-fat milk for 1 h and incubated with the following primary antibodies overnight at 4 °C: anti-Bax (1:1000, Abcam), anti-Bcl-2 (1:1000, Abcam), anti-cleaved caspase-3 (1:1000, Abcam), anti-GSDME-N (1:1000, Abcam), anti-cleaved caspase-1 (1:1000, Abcam), anti-p-EGFR (1:1000, Abcam), anti-EGFR (1:1000, Abcam), and anti-β-actin (1:1000, Abcam). The following day, the membranes were incubated with the horseradish peroxidase-conjugated IgG secondary antibody (1:5000, Abcam) at room temperature for 1 h. Finally, the blots were visualized using the ECL chemiluminescent substrate reagent (Thermo Fisher Scientific).

### Dual-luciferase reporter assay

The 3′-UTR sequences of GSDME that contained the predicted wild-type (WT) or mutant (MT) miR-155-5p binding sequences were ligated into the pGM-CMV-Luc vector (Yeasen, Shanghai, China). pGM-CMV-Luc-WT-GSDME-3’-UTR or pGM-CMV-Luc-MT-GSDME-3’-UTR was co-transfected into cells with the miR-155-5p agomir using Lipofectamine 2000. Luciferase activity was detected using the Dual-Luciferase Reporter Assay System (Promega, Madison, WI, USA) at 48 h, and Renilla luciferase activity was used as the endogenous control.

### Transmission electron microscopy

The ultrastructure of the cells was observed with a transmission electron microscope (TEM, H-600IV, Hitachi Ltd., Japan). Briefly, the cells were fixed in 2.5% glutaraldehyde (GA, Sigma-Aldrich, St. Louis, MO, USA) at 4 °C overnight and dehydrated in ethanol. The samples were captured using a TEM as described previously [35].

### Animal study

Four to 6-week-old BALB/c nude mice were purchased from the Hubei Provincial center for Laboratory Animal and maintained following the guidelines of the Institutional Animal Care and Use Committee. Animals were randomized into four groups: 1) Vehicle, 2) cetuximab, 3) miR-155-5p antagomir, and 4) cetuximab + miR-155-5p antagomir group. EGFR-overexpressed MDA-MB-468 cells (1 × 10^7^ per mouse in 100 μL of PBS) were subcutaneously injected into the left flank of nude mice. When the tumors reached approximately 200 mm^3^, 50 nM miR-155-5p antagomir was directly injected into the tumors twice weekly, and the mice received weekly intraperitoneal injections of cetuximab (50 mg/kg). Tumor volume was measured every week using the following formula:

V = (length x width^2^)/2 (width < length)

The mice were euthanized at 28 days after treatment, and the tumors were isolated and weighed**.** All of the animal experiments were approved by the Institutional Ethical Committee of Longhua Hospital Affiliated to Shanghai University of TCM.

### IHC analysis

IHC assays were performed to determine the expression level of Ki67 in tumor tissues. Tissue specimens were sectioned into 5 μm thick slices and incubated with the primary Ki67 antibody overnight at 4 °C. The following day, the tissue was incubated with biotinylated goat anti-rabbit IgG for 30 min at room temperature. IHC reactions were visualize using the IHC detection system (EnVision kit; Dako Japan).

### Statistical analysis

All data were repeated in triplicate. Data are presented as the mean ± standard deviation (S.D.). All statistical analyses were performed using GraphPad Prism software (version 7.0, La Jolla, CA, USA). One-way analysis of variances (ANOVAs) were performed for multiple group comparisons, and pairwise comparisons were conducted with Tukey’s tests when applicable. *P < 0.05 was considered to be statistically significant.
